# HmuY Haemophore and Gingipain Proteases Constitute a Unique Syntrophic System of Haem Acquisition by *Porphyromonas gingivalis*

**DOI:** 10.1371/journal.pone.0017182

**Published:** 2011-02-17

**Authors:** John W. Smalley, Dominic P. Byrne, Andrew J. Birss, Halina Wojtowicz, Aneta Sroka, Jan Potempa, Teresa Olczak

**Affiliations:** 1 School of Dental Sciences, The University of Liverpool, Liverpool, United Kingdom; 2 Laboratory of Biochemistry, Faculty of Biotechnology, University of Wroclaw, Wroclaw, Poland; 3 Department of Microbiology, Faculty of Biochemistry, Biophysics and Biotechnology, Jagiellonian University, Krakow, Poland; 4 Department of Oral Health and Rehabilitation, University of Louisville School of Dentistry, Louisville, Kentucky, United States of America; University of Delhi, India

## Abstract

Haem (iron protoporphyrin IX) is both an essential growth factor and virulence regulator for the periodontal pathogen *Porphyromonas gingivalis*, which acquires it mainly from haemoglobin via the sequential actions of the R- and K-specific gingipain proteases. The haem-binding lipoprotein haemophore HmuY and its cognate receptor HmuR of *P. gingivalis*, are responsible for capture and internalisation of haem. This study examined the role of the HmuY in acquisition of haem from haemoglobin and the cooperation between HmuY and gingipain proteases in this process. Using UV-visible spectroscopy and polyacrylamide gel electrophoresis, HmuY was demonstrated to wrest haem from immobilised methaemoglobin and deoxyhaemoglobin. Haem extraction from oxyhaemoglobin was facilitated after oxidation to methaemoglobin by pre-treatment with the *P. gingivalis* R-gingipain A (HRgpA). HmuY was also capable of scavenging haem from oxyhaemoglobin pre-treated with the K-gingipain (Kgp). This is the first demonstration of a haemophore working in conjunction with proteases to acquire haem from haemoglobin. In addition, HmuY was able to extract haem from methaemalbumin, and could bind haem, either free in solution or from methaemoglobin, even in the presence of serum albumin.

## Introduction

*Porphyromonas gingigivalis* is a gram-negative oral anaerobe which has been implicated as one of the major aetiologic agents of periodontitis in adults [1]. It displays a black-pigmenting phenotype which is due to accumulation of a haem-containing pigment. The pigment is formed during growth on blood containing media and the haem component of this is derived from proteolytic breakdown of the haemoglobin, and the haem-carrying proteins haemopexin and haemalbumin. The haem in the pigment is present in the so-called µ-oxo bishaem, or dimeric form of iron(III) protoporphyrin IX, [Fe(III)PPIX]_2_O, which is stored in aggregated form on the *P. gingivalis* cell surface [2]. *P. gingivalis* has an essential requirement for haem [3] which has a profound influence on its virulence properties [4], [5]. However, *P. gingivalis* cannot synthesize this iron porphyrin and thus acquisition from exogenous sources, coupled with its binding and cell surface storage, is essential for the growth and survival of this bacterium. Whilst the agent(s) responsible for haem deposition at the cell surface have not been elucidated, binding of haem for purposes of internalisation is mediated by a dual component uptake system comprising the haem-binding protein HmuY and its cognate receptor HmuR [6]. Both HmuY and HmuR are encoded by the *hmu* operon, encompassing a total of six genes, *hmuYRSTUV*, which is regulated by the environmental levels of iron [7]. The potential importance of the *hmu* operon in haem acquisition has been demonstrated by the fact that its inactivation results in reduction of both cellular haem binding and its uptake [8]. The mature membrane-associated HmuY protein has a molecular weight of 24 kDa [9]. As a non-haem liganded apoprotein, it exists as a homodimer, whereas upon binding of haem it can form a homo-tetrameric complex [7], [9]. HmuY can also bind iron protoporphyrin IX in both Fe(III) and Fe(II) states [10]. Point mutation studies have shown that residues His134 and His166 of HmuY are involved in six-coordinate binding of haem [10]. Concurrently, solution of the crystal structure of HmuY has confirmed this arrangement and demonstrated a unique all beta-fold protein structure [9].

The emerging paradigm for proteolytically mediated haem acquisition from oxygenated haemoglobin involves the concerted action of both arginine- and lysine-specific gingipain proteases, Rgp and Kgp, [11], [12], [13]. In this process the Fe(II) oxyhaemoglobin species is firstly oxidised by the proteolytic action of Rgp giving rise to methaemoglobin, the oxidised form of haemoglobin. The concomitant reduction in the affinity of globin for the iron protoporphyrin IX, now in the Fe(III) state, results in dissociation of the haem moiety from the globin protein, which is then rendered susceptible to degradation by Kgp [12], [13]. It is well documented that the clearance of free haem from the systemic circulation occurs following oxidation of oxyhaemoglobin, and the ferric haem thus formed is easily transferred from methaemoglobin to albumin [14], [15]. Given the propensity for more facile haem loss and the proteolytic susceptibility of ferric haemoglobins, together with the demonstrated affinity of HmuY for ferric (and ferrous) haem species [7], [10], we thus investigated the role of this haemophore protein in acquiring haem directly from haemoglobin and also the cooperative role of the *P. gingivalis* gingipain proteases in this process.

## Results

### Formation of ferrihaem-HmuY and ferrohaem-HmuY

**Figure 1:**
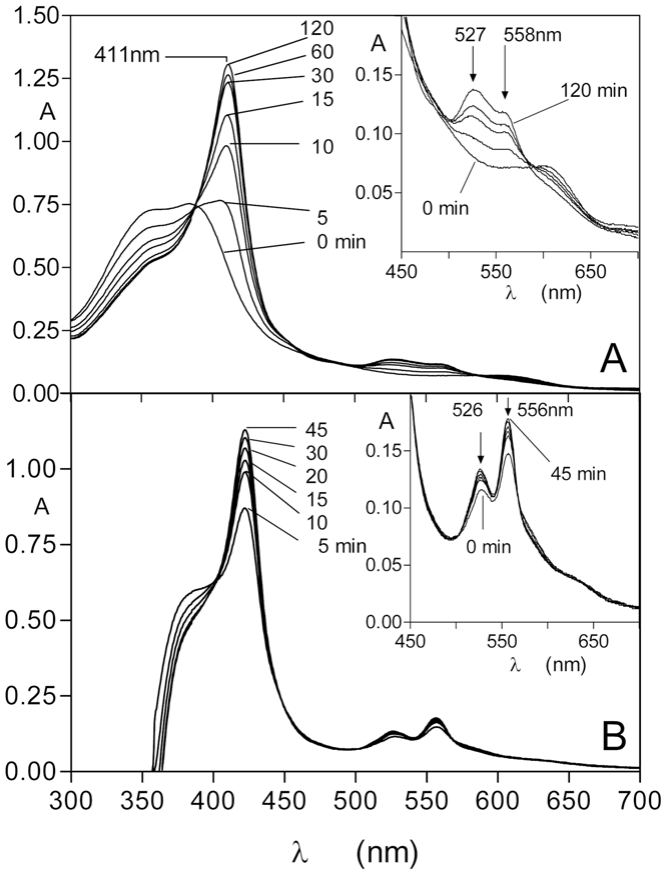
Formation of the HmuY-ferrihaem (A) and HmuY-ferrohaem (B) complexes during reaction of HmuY with iron protoporphyrin IX. HmuY (16 µM) was reacted with an equimolar amount of iron protoporphyrin IX. In (B) the ferrohaem species was generated by inclusion of 10 mM Na_2_S_2_O_4_ in the buffer. The steep drop in absorbance below 375 nm is due to subtraction of the reference background spectrum of the dithionite-containing buffer.

To confirm the spectrum of the Fe(III) haem-HmuY complex, the protein was incubated at 37°C with an equimolar quantity of iron(III) protoporphyrin IX in NaCl-Tris-HCl buffer, pH 7.5. As seen in Figure 1, the HmuY reacted rapidly with the iron(III) protoporphyrin IX causing a red shift simultaneous with the increase of the extinction of the Soret band (λ_max_ 411 nm), plus Q bands of approximately 527 nm and 558 nm. This is in accord with the previously published spectrum for the six-coordinate, bis-histidine ligated, 1∶1 HmuY-ferrihaem low-spin complex [10]. The generation of the HmuY monomeric ferrihaem complex under these conditions reached a maximum after approximately 120 min, after which time no further changes to the spectrum were observed. The series of time-course spectra depicted in Fig. 1A displayed an isosbestic point at approximately 388 nm showing the presence of only two absorbing species and indicating the direct conversion of the free ferrihaem into the HmuY-iron(III) protoporphyrin IX complex. This is in keeping with the known 1∶1 stoichiometry of this interaction [7]. The binding of iron protoporphyrin IX was also monitored in the presence of 10 mM Na_2_S_2_O_4_ to reduce the iron(III) protoporphyrin IX to the Fe(II) species (Fig. 1B). Reaction of the HmuY with this form of haem gave the Fe(II) haem-HmuY complex with Soret band λ_max_ of 422 nm and Q bands at 526 nm and 556 nm, consistent with the previously published spectrum of the ferrohaem-HmuY complex [10]. Under these conditions, the ferrohaem-HmuY complex formed immediately upon mixing and the Soret band attained a maximum after 45 min after which time there was no further change in the spectrum.

### Effect of haem complexation on the electrophoretic mobility of HmuY

**Figure 2:**
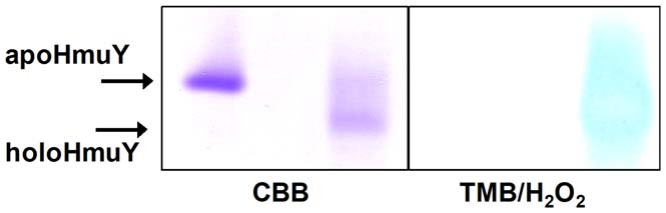
Effect of ferrihaem binding on the electrophoretic mobility of HmuY. The presence of haem in the complex with HmuY is confirmed by haem-peroxidise activity (bottom panel).

Complexation of HmuY with an equimolar amount of ferrihaem increased significantly the electrophoretic mobility of the HmuY as analyzed by the native PAGE (Fig. 2). This effect is presumed to occur as a consequence of bis-histidine ligation of haem by the HmuY, resulting in a haem-HmuY complex in which the ionised carboxylate groups of the iron porphyrin impart additional negative charge, which makes the liganded holoHmuY migrate faster than apoHmuY in the electric field during native PAGE.

### HmuY binds iron(III) protoporphyrin IX from methaemoglobin

**Figure 3:**
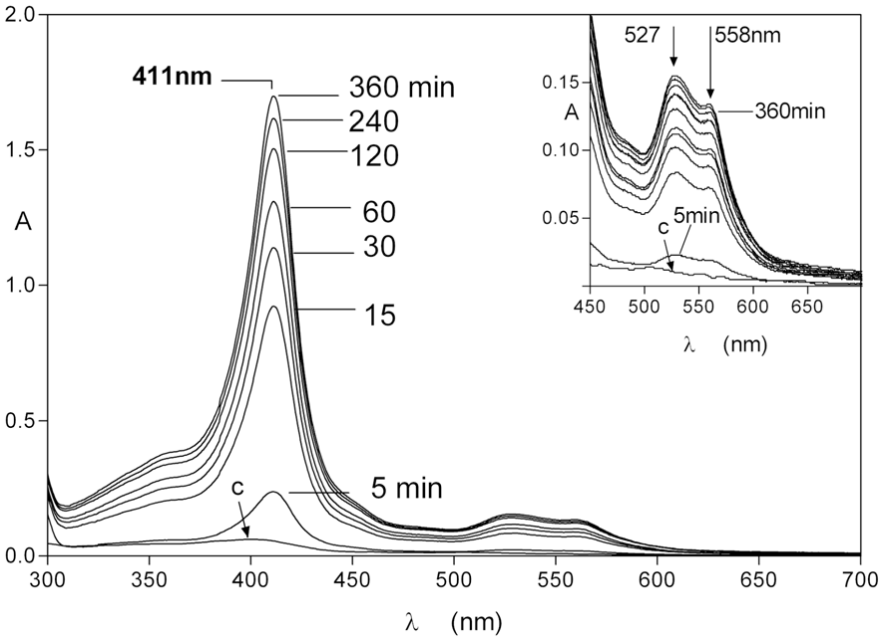
Spectroscopic demonstration of the formation of the HmuY-ferrihaem complex following interaction with immobilised methaemoglobin. Methaemoglobin-agarose (16 µM with respect to haemoglobin subunit) was incubated with an equimolar amount of HmuY and at various time periods the agarose beads sedimented by centrifugation and the spectra of the supernatant recorded. The spectrum denoted c represents the small amount of methaemoglobin (∼0.25% of total) spontaneously released from the control methaemoglobin-agarose beads after 6 h in the absence of HmuY. The incubations were carried out at 37°C. See text for details.

To determine whether the haemophore could form a ferrihaem complex through interaction with methaemoglobin, HmuY was incubated with immobilised methaemoglobin conjugated to agarose. Such preparations have been used previously to determine the rates of haem loss from haemoglobin [16]. This method allows the monitoring of protein-haem complex formation (in solution) separately from the haemoglobin attached to the solid phase in a way free of any compounding spectral features due to the co-presence of haemoglobin. Previously we have used this approach to demonstrate the facile breakdown and release of haem from methaemoglobin by Kgp [13] and interpain A of *Prevotella intermedia* [17]. Here we incubated HmuY with methaemoglobin-agarose and at various time intervals sedimented the beads by low speed centrifugation, and recorded the spectrum of the agarose-free supernatant. The spectral analyses revealed a typical ferrihaem-HumY spectrum with the characteristic 411 nm Soret band and 527 and 558 nm visible bands which developed within 5 minutes of mixing (Fig. 3). Following the addition of 10 mM sodium dithionite to the incubation mixture a spectrum indicative of the ferrohaem-HmuY complex developed (a 422nm Soret and 526 and 556 nm Q bands) (data not shown).

**Figure 4:**
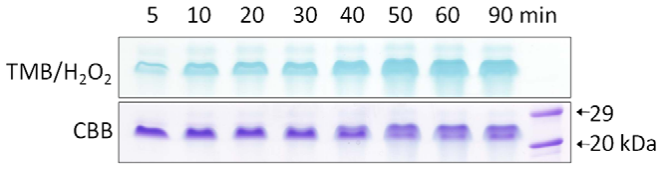
Haem pickup by HmuY from methaemoglobin-agarose. HmuY (16 µM) was incubated with methaemoglobin-agarose beads and after centrifugation the agarose-free supernatant was sampled and analyzed by non-reducing SDS-PAGE, and stained with TMB/H_2_O_2_ to reveal haem-associated peroxidise activity before being stained with Coomassie brilliant blue (CBB). Protein loading per track was 7 µg.

The results of spectral analysis indicating HmuY-haem complex formation were corroborated by non-reducing SDS-PAGE analysis of samples obtained at different time intervals during incubation of HmuY with immobilized methaemoglobin. The HmuY protein band showed a time-dependent increase in TMB/H_2_O_2_ haem-peroxidase staining unequivocally confirming the formation of the HmuY-haem complex (Fig. 4).

### Methaemoglobin formation by R-gingipain facilitates extraction of haem from haemoglobin by HmuY

**Figure 5:**
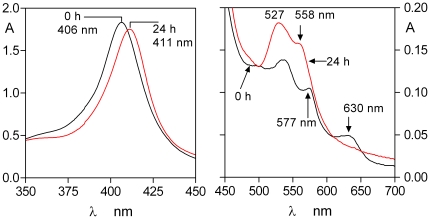
Demonstration HmuY-ferrihaem complex formation after exposure of HRgpA-induced methaemoglobin to HmuY. Before exposure, black line; 24 h after exposure, red line. For clarity, only the initial and final spectra are shown. HRgpA and HmuY were used at 0.4 and 16 µM, respectively. Starting concentration of haemoglobin was 16 µM (with respect to haemoglobin subunit) and comprised 77% methaemoglobin.

*In vivo*, at periodontitis sites where bleeding may occur, any oxyhaemoglobin released from erythrocytes must firstly be oxidized to the methaemoglobin form before haem can be sequestered by HmuY. Therefore the process of haem acquisition and uptake by the HmuY/HmuR system should be dependent upon gingipain protease-mediated haemoglobin oxidation [12], [13]. To experimentally verify this hypothesis, an HRgpA-induced methaemoglobin preparation (total haemoglobin concentration 16 µM and comprising 77% in the oxidised form) was incubated with HmuY (16 µM). A control NaNO_2_-induced methaemoglobin preparation (containing 83% in the oxidised form) was also incubated with HmuY as above for comparison. With time, spectral features were observed indicative of conversion of R-gingipain-induced methaemoglobin into the ferrihaem-HmuY complex, i.e., red shift of the Soret band from 406 to 411 nm, loss of A_500 nm_ and A_630 nm_, and increases in A_527 nm_ and A_558 nm_, as shown in Fig. 5. These spectral features were also observed for the incubation of HmuY with the NaNO_2_-induced methaemoglobin preparation (data not presented).

To confirm that haem had been complexed by HmuY and was not still present in the form of haemoglobin, the reaction mixture was treated with 10 mM Na_2_S_2_O_4_ and the UV-visible spectrum recorded. Analysis of this spectrum showed a 423 nm Soret band, plus visible bands at 526 nm and 556 nm, clearly indicating the presence of the ferrohaem-HmuY complex (data not presented). This result unambiguously indicated that haem had become complexed to HmuY during incubation with the methaemoglobin which had been formed by the action of HRgpA.

**Figure 6:**
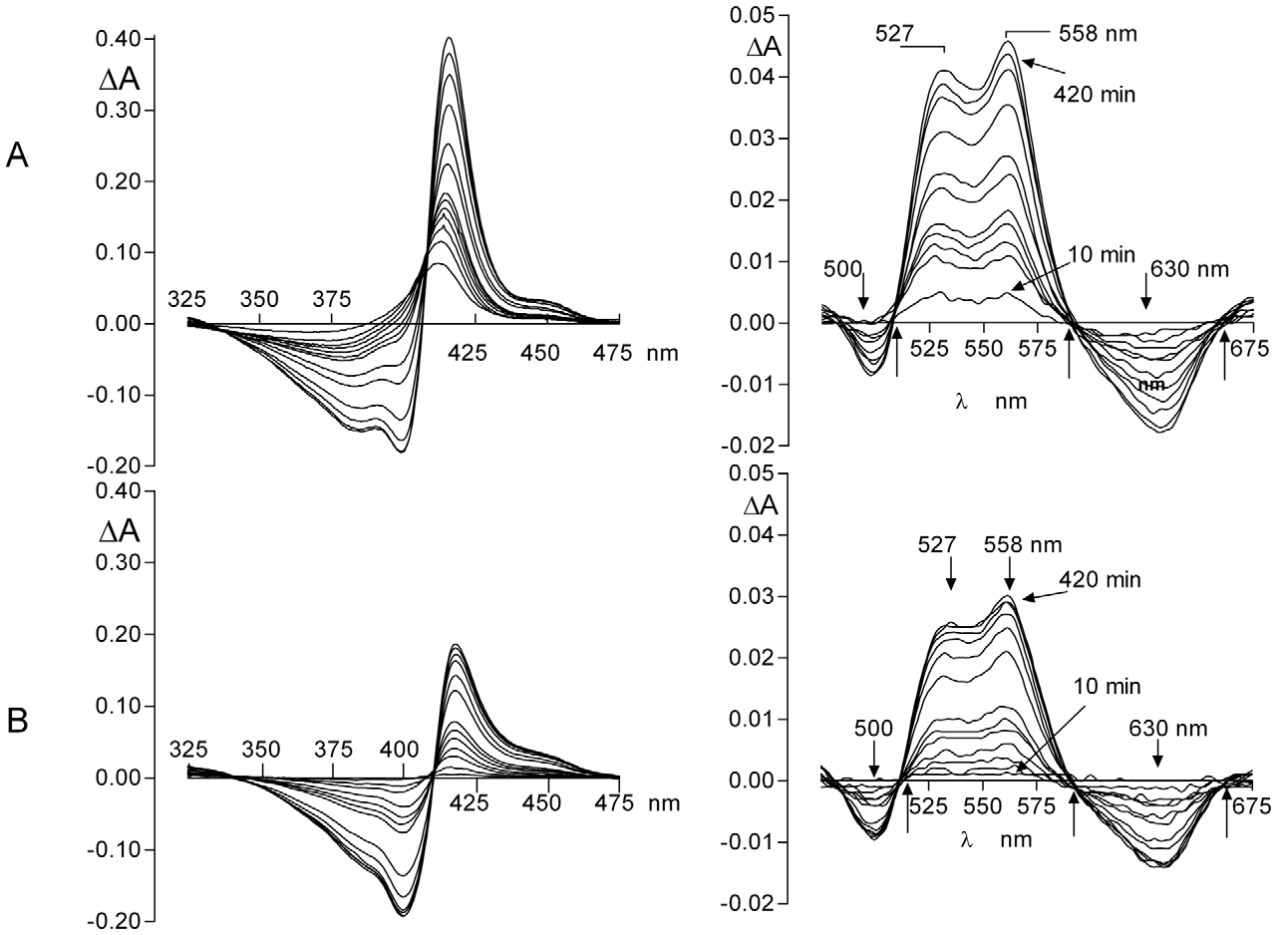
Difference spectra showing HmuY-haem complex formation from HRgpA-methaemoglobin (A) and NaNO_2_-induced methaemoglobin (B). The difference spectra in the visible region show the appearance of the 525 nm and 558 nm bands attributable to formation of the HmuY-ferrihaem complex. Note that the relative extinctions at 527 nm and 558 nm are reversed compared to those in the ferrihaem-HmuY complex shown in Fig. 1, and occur as a result of subtraction of the spectra. Starting concentrations of haemoglobin were 16 µM (haemoglobin subunit basis) and contained 77 and 87% methaemoglobin for the HRgpA- and NaNO_2_-treated oxyhaemoglobin, respectively. HRgpA and NaNO_2_ concentrations were 0.4 µM and 64 µM, respectively.

The facile transfer of iron(III) protoporphyrin IX from methaemoglobin to HmuY was further corroborated by assessment of the relative amount of the HmuY-ferrihaem complex formed from either HRgpA- or NaNO_2_-induced methaemoglobin. To this end, difference spectra were derived by subtracting the control spectra from the tests at each time interval (Fig. 6A and B) which showed that the amount of HmuY-haem complex formed was greater from methaemoglobin proteolytically induced by HRgpA compared to that produced by NaNO_2_ treatment.

These difference spectra were also characterised by troughs at 630 nm and 500 nm, which related to loss of methaemoglobin, and increases in absorbance at 525 nm and 558 nm, attributable to the formation of the HmuY-ferrihaem complex. Isosbestic points were observed at 510 nm, 590 nm, and 660 nm (arrowed) showing the direct conversion from methaemoglobin to HmuY-haem complex. These spectra clearly showed that the amount of HmuY-haem complex formed from HRgpA-pre-treated haemoglobin was greater than methaemoglobin formed by treatment with NaNO_2_.

### HmuY-haem complex forms during co-incubation of oxyhaemoglobin with both HRgpA and HmuY

**Figure 7:**
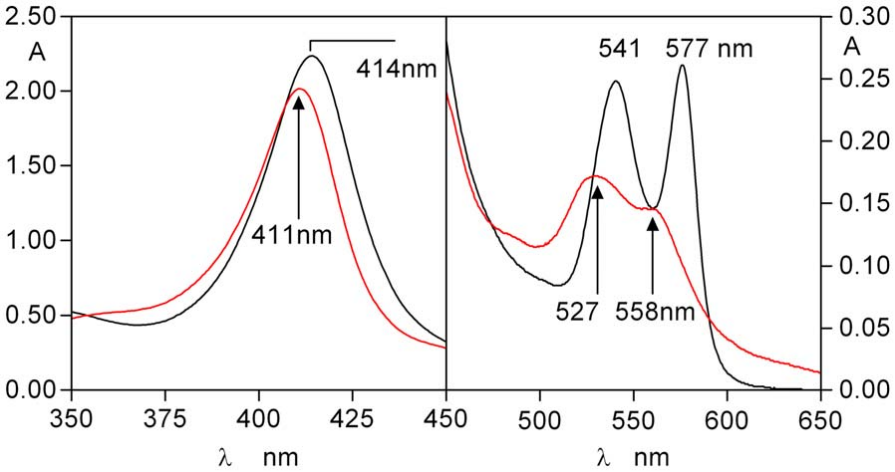
Spectrum showing the ferrihaem-HmuY complex (red line) formed after 24 h co-incubation of oxyhaemoglobin with HRgpA and HmuY. The oxyhaemoglobin and HmuY concentrations were both 16 µM, whilst HRgpA was at 0.4 µM.

**Figure 8:**
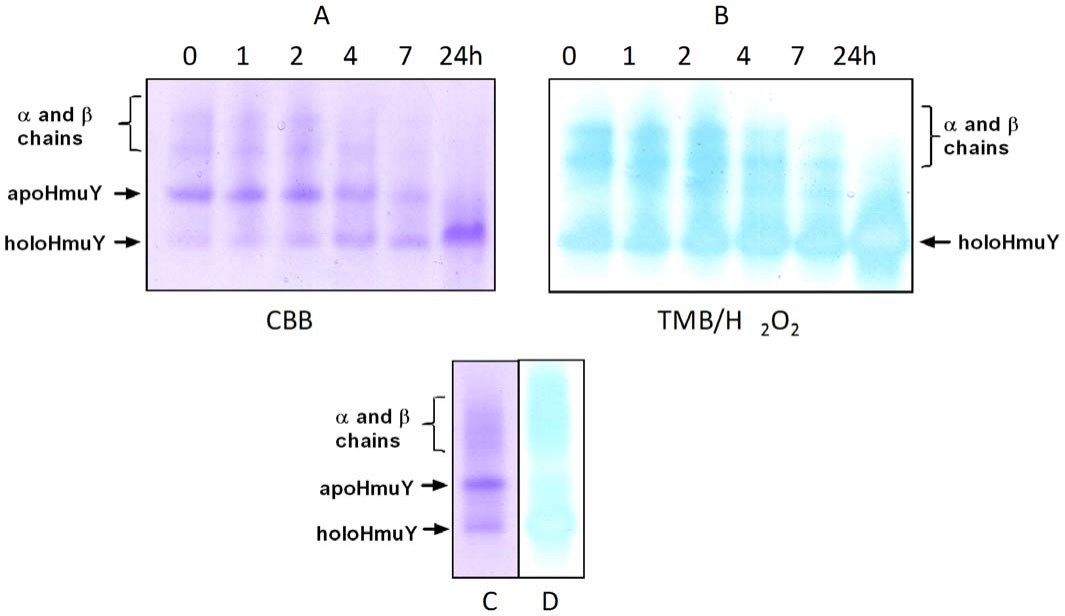
Native PAGE showing the HmuY-haem complex formed through co-incubation of oxyhaemoglobin with HRgpA plus HmuY. Oxyhaemoglobin was co-incubated with HmuY plus HRgpA (CBB stained gel A and TMB/H_2_O_2_ stained gel B). Note that a small amount of the HmuY-haem complex was formed after 24 h incubation of oxyhaemoglobin plus HmuY only (CBB stained gel track C and TMB/H_2_O_2_ stained gel track D), attributed to pickup of haem lost from oxyhaemoglobin as a result of auto-oxidation. The oxyhaemoglobin concentration was 16 µM (on a haemoglobin subunit basis), as was HmuY, whilst HRgpA was present at 0.4 µM.

Chemostat studies [3] have shown the presence of arginine-specific protease activity in haem-limited cultures expressing haem-binding proteins [4], [18]. Since *P. gingivalis* may concomitantly deploy the HmuY haemophore and gingipain proteases, we investigated the effect of co-incubation of oxyhaemoglobin with HmuY in the presence of HRgpA to mimic the *in vivo* situation more closely. It is noteworthy in this respect, that HmuY is absolutely resistant to degradation by either R- or K-gingipains [9]. For this, HmuY (16 µM) was incubated with 16 µM oxyhaemoglobin (with respect to haemoglobin subunit) in the presence of HRgpA (400 nM) and the haem transfer from oxyhaemoglobin to HmuY monitored spectroscopically and by native PAGE (Figs. 7 and 8, respectively). In the presence of both HRgpA and HmuY, the oxyhaemoglobin spectrum was transformed after 24 h into one typical of the HmuY-ferrihaem complex with a 411 nm Soret band and 528 nm and weak 559 nm visible bands (Fig. 7).

**Figure 9:**
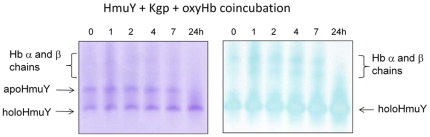
HmuY-haem complex formation during the co-incubation of oxyhaemoglobin with both HmuY and Kgp. HmuY and oxyhaemoglobin (both at 16 µM) were incubated at 37°C with Kgp (0.2 µM) and sampled periodically, and subjected to native PAGE. Gel tracks were loaded with ∼12 µg total protein.

During electrophoresis under non-denaturing conditions, apoHmuY migrated as a single band with an R_f_ greater than that of the α and β haemoglobin chains (Fig. 9). During incubation of oxyhaemoglobin with HRgpA plus HmuY there was a progressive increase in the degree of CBB staining of the faster migrating holoHmuY (Fig. 8A; arrowed) which was matched by an increase in the TMB/H_2_O_2_ staining intensity (Fig. 8B). This was accompanied by a reciprocal decrease in both the TMB/H_2_O_2_ and CBB staining of the slower migrating haemoglobin bands, the latter being typical of haem-free globin chains [13]. It is noteworthy that a small amount of the faster moving HmuY-haem complex was also generated (most notably after 24 h) when oxyhaemoglobin was incubated only with HmuY (Fig. 8, gel tracks C and D). This was attributed to the HmuY pickup of haem lost from oxyhaemoglobin as a result of auto-oxidation during the period of the experiment.

### *P. gingivalis* Lys-gingipain (Kgp) also facilitates haem acquisition by HmuY

It has been previously demonstrated that the proteolytic attack by Kgp on oxyhaemoglobin results in the formation of a haemoglobin haemichrome, stable to further degradation by the enzyme [12]. Because of the close similarities of the spectra of the haemoglobin haemichrome (409 nm Soret and 535 nm visible bands) and the HmuY-ferrihaem complex, a definitive identification of the formation of the latter from the product of oxyhaemoglobin proteolysis by Kgp is not possible by UV-visible spectroscopy alone. Therefore, the co-incubation mixtures of oxyhaemoglobin in the presence of both Kgp and HmuY were examined as above by non-denaturing PAGE. This showed both an increase in the mobility and TMB/H_2_O_2_ haem staining of the HmuY (Fig. 9). Indeed, there was demonstrable TMB/H_2_O_2_ staining of the faster-migrating liganded HmuY as soon as the mixing had taken place (at time 0), clearly showing the facile haem pick up by the HmuY from the haemichrome.

### HmuY mediates ferrohaem pickup from deoxyhaemoglobin

**Figure 10:**
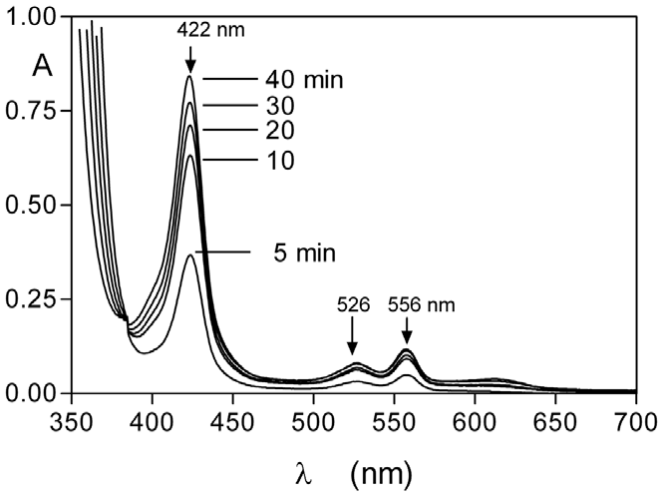
Formation of the ferrohaem-HmuY complex during incubation of HmuY with deoxyhaemoglobin-agarose. Haemoglobin-agarose was deoxygenated with sodium dithionite and maintained anaerobically during the reaction with HmuY. Concentration of haemoglobin was 16 µM (with respect to haemoglobin subunit) as was HmuY. See text for details. The absorbance below 375 nm is due to the presence of dithionite. A small amount of deoxyhaemoglobin (∼0.1% of the total) was released from the control deoxyhaemoglobin-agarose after 40 min incubation in the absence of HmuY.

Since *P. gingivalis* may also encounter deoxygenated haemoglobin in the anaerobic gingival sulcus or periodontal pocket, it was decided to investigate the interaction of HmuY with deoxyhaemoglobin-agarose. The haemoglobin-agarose was deoxygenated by the addition of sodium dithionite [19] and maintained anaerobically whilst periodically removing the agarose-free, HmuY-containing supernatants for which the spectra were recorded. These showed that a ferrohaem-HmuY complex developed within 5 min of exposure of the deoxyhaemoglobin to HmuY (Fig. 10).

### HmuY can extract haem from methaemoglobin in the presence of serum albumin

**Figure 11:**
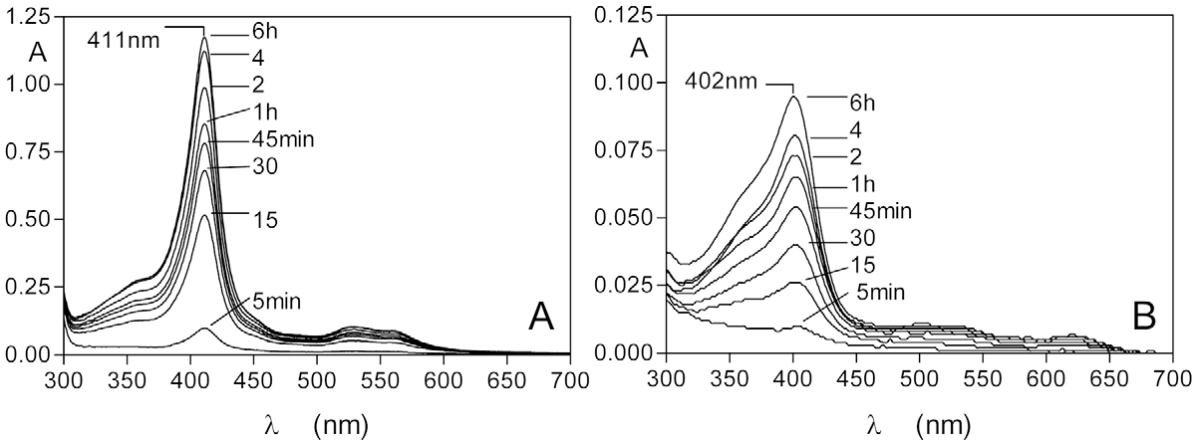
Formation of the HmuY-ferrihaemcomplex during incubation of methaemoglobin-agarose in the presence of human serum albumin. HmuY, albumin and methaemoglobin were each present at 16 µM (with the haemoglobin on a subunit basis). Panel A, methaemoglobin-agarose co-incubated with both HmuY and albumin. Panel B, methaemoglobin plus albumin only. The experimental protocol was the same as described for Fig. 4, and the incubations were carried out at 37°C. The spectra are background corrected to take account of the small amount of methaemoglobin released at each time period from the haemoglobin agarose during incubation.

We tested whether HmuY was capable of binding haem complexed in the form of methaemoglobin but in the presence of serum albumin. It should be noted that methaemalbumin and methaemoglobin share similar spectral features at physiological pH and that the presence of all three components in solution would yield a very complex spectrum. Therefore, we used immobilised methaemoglobin-agarose as the substrate so that the differential formation of either the methaemalbumin or HmuY-ferrihaem complexes (which are spectrally distinct) could be more easily followed. All the three components each at 16 µM (with methaemoglobin on a subunit basis as above) were incubated together at 37°C, and the supernatant solutions were removed periodically from the agarose beads, and the spectra recorded as for the two component (HmuY/methaemoglobin-agarose) system. As can be seen in Fig. 11 (panel A), in the three component mixture a spectrum was observed with a 411 nm Soret band and visible bands at 527 and 558 nm, which was indicative of the formation of the HmuY-ferrihaem species. The intensities of these bands increased with time of incubation as previously demonstrated in Figure 4 for the reaction of HmuY plus methaemoglobin-agarose only. In contrast however, for the incubation of the methaemoglobin-agarose with albumin generated spectra with a 402 nm Soret band and lower intensity bands at 500 and 620 nm, indicative of the formation of the methaemalbumin complex (Fig. 11, panel B).

### HmuY binds free ferrihaem in the presence of serum albumin

**Figure 12:**
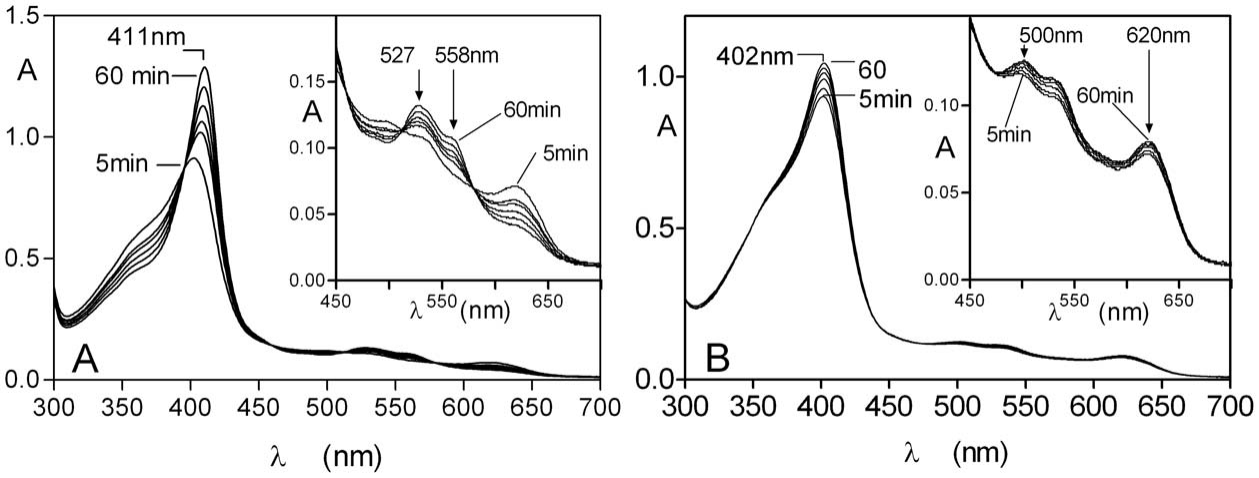
Spectroscopic demonstration of the formation of the HmuY-ferrihaem complex during co-incubation of HmuY and iron(III) protoporphyrin IX in the presence of human serum albumin. Panel A, spectra obtained during incubation of all three components. Panel B, incubation of albumin and haem. All components were present at 16 µM. See text for details.

To determine whether HmuY could also compete with serum albumin for free iron(III) protoporphyrin IX, a mixture of both proteins (each at 16 µM) was incubated with haem (also at 16 µM) at 37°C and the spectra were recorded periodically. As seen in Fig. 12A, there was an immediate red shift in the Soret band from a λ_max_ of 385 nm (that of haem) to 411 nm, and the appearance of visible bands at 527 and 558 nm as observed for the addition of haem to HmuY as seen in Fig. 1 for the formation of the HmuY-ferrihaem complex. Note the presence of isosbestic points at 395, 460, 512 and 580 nm indicating the direct conversion of the free haem into the HmuY-ferrihaem complex. In contrast, incubation of serum albumin with ferrihaem resulted in the formation of a Soret band with λ_max_ of 402 nm, indicative of the presence of methaemalbumin (Fig. 12B).

### HmuY can extract haem from methaemalbumin

**Figure 13:**
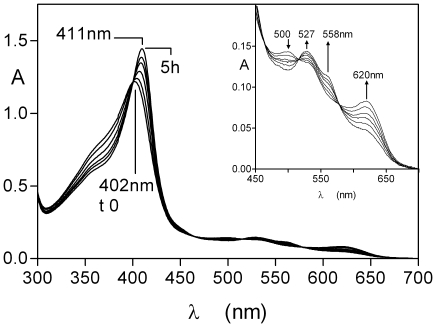
Formation of the ferrihaem-HmuY complex during incubation of human methaemalbumin with HmuY. Methaemalbumin (16 µM) was incubated with an equimolar amount of HmuY at 37°C. Arrows denote changes in the spectra with time at the indicated wavelengths.

In the absence of free haem or that in the form of methaemoglobin, the HmuY haemophore may also have to compete for haem already complexed by serum haem-binding proteins. We therefore tested this by incubating methaemalbumin (16 µM) at 37°C with an equimolar quantity of HmuY and periodically monitored the UV-visible spectrum. As seen in Fig. 13, there was a progressive increase in the intensity and red shift in the Soret band from 402 nm to 411 nm. This was accompanied by a decrease in the 500 and 620 nm methaemalbumin associated bands, and a reciprocal increase in the absorbance at 527 nm with a shoulder at 558 nm band, features indicative of the formation of the HmuY-ferrihaem complex. Clear isosbestic points were observed at 464, 515, and 578 nm indicating that the methaemalbumin had been transformed directly into the HmuY-ferrihaem complex.

**Figure 14:**
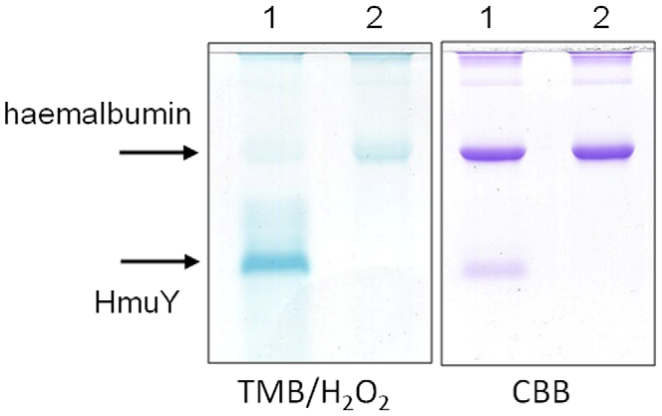
SDS-PAGE showing the formation of the HMuY-haem complex after incubation with human methaemalbumin. Methaemalbumin (16 µM) was with either 16 µM HmuY (track 1) or alone (track 2) for 5 h at 37°C, electrophoresed under non-reducing and then stained for the presence of haem with TMB/H_2_O_2_ before counterstaining for protein with CBB. Note the depletion of TMB/H_2_O_2_ staining for haem of the methaemalbum band in track 1 after exposure to HmuY.

To confirm the presence of the HmuY-haem complex, a sample of the incubation mixture (after 5 h) was subjected to non-reducing SDS-PAGE and the proteins stained for the presence of haem with TMB/H_2_O_2_, and then counterstained with CBB (Fig. 14). As can clearly be seen, the methaemalbumin complex (track B) had been almost completely depleted of haem as revealed by TMB/H_2_O_2_ staining, whilst in contrast the faster moving HmuY band was heavily stained for haem.

## Discussion

We have demonstrated in this study that, in addition to binding free haem, the HmuY haemophore of *P. gingivalis* can extract ferrihaem from methaemoglobin. This is a significant advancement in the understanding of the mechanisms of haem acquisition by this organism. The paradigm for proteolytically-mediated haem acquisition from oxyhaemoglobin by *P. gingivalis* involves its initial oxidation to the methaemoglobin form by the action of R-gingipain [11], [12], [13]. This results in the relaxation of the affinity of globin for the ferrihaem species (compared to that of iron(II) haem). As a consequence, this greatly facilitates the proteolytic attack on the globin protein by Kgp and subsequent haem release [12], [13]. In this respect it is noteworthy that in the body reprocessing of haemoglobin from damaged and senescent erythrocytes firstly involves the oxidation of haem such that it can be removed by either serum albumin and/or haemopexin which have association constants for ferrihaems of 10^8^ and 2×10^14^ M^−1^, respectively [20], [21]. Thus, in parallel with this paradigm was the finding that methaemoglobin formed by the proteolytic attack on oxyhaemoglobin by HRgpA was a facile “substrate” from which the HmuY molecule could easily remove haem. To our knowledge, this is the first demonstration of a bacterial haemophore acting syntrophically with proteases to extract haem from haemoglobin.

We have previously demonstrated the presence of a haem-binding protein in chemostat cultures of *P. gingivalis* run under conditions of both haem excess and haem limitation [18]. Under these conditions, these cells produce arginine-specific protease activity [4]. In addition, Olczak *et al.* [22] have shown that HmuY is expressed at higher levels by cells in batch culture in which the haem level is restricted. Therefore it is likely *in vivo* that *P. gingivalis* would concomitantly deploy a combination of protease and haemophore molecules as a syntrophic haem acquisition mechanism system. Importantly, in addition, it has been demonstrated that HmuY is completely resistant to the proteolytic effects of HRgpA, RgpB and Kgp [9], indicating that HmuY could very well function alongside these proteases in the process of haem acquisition.

The primary habitat of *P. gingivalis* and other black-pigmenting anaerobes is the diseased periodontal pocket and gingival sulcus. These environments have been shown to be slightly alkaline [23], [24]. The need for *P. gingivalis* to rely upon proteolytically–mediated oxidation of oxyhaemoglobin as a first step in haem removal from oxyhaemoglobin in this type of environment is critically important. This stems from the fact that at alkaline pH, the natural oxidation rate of oxyhaemoglobin is at its lowest [25]. Thus, haem acquisition by *P. gingivalis* from oxyhaemoglobin reaching this environment as a result of bleeding and haemolysis, would be reliant upon a mechanism which would promote its oxidation. In this regard, it is noteworthy that gingipains have an alkaline pH optimum [26], [27].

Although diseased periodontitis sites may experience fresh bleeding as a result of inflammation and micro-trauma, very anaerobic and reduced gingival sulcus and pocket microenvironments are also likely to contain deoxygenated haemoglobin. In view of this likelihood, we also investigated the pickup of ferrohaems from deoxyhaemoglobin-agarose which was maintained anaerobically. We unequivocally demonstrated the rapid generation of the ferrohaem-HmuY complex immediately after mixing of the two proteins. Importantly, whilst this compares starkly with the difficulty of haem pick up from the oxygenated protein (in the absence of the prior oxidation to the met-form), it nevertheless clearly shows the versatility of the HmuY haemophore which has the ability to extract haems from both the deoxy-iron(II) as well as from the oxidised haemoglobin species.

We have found that whilst Kgp is effective in degrading methaemoglobin, especially that produced by the action of HRgpA on oxyhaemoglobin [12], [13], the action of Kgp on oxyhaemoglobin results in the formation of a haemoglobin haemichrome which is resistant to further attack by the Kgp protease [12], and also by HRgpA (Smalley JW, Birss AJ, and Potempa J, unpublished findings). However, this study has shown that haem can be extracted by HmuY from the haemoglobin haemichrome and has revealed yet another route through which haem might be acquired as a consequence of the deployment of the gingipain proteases.

The question arises as to the capability of HmuY of gaining haem in the inflamed gingival crevice and periodontal pocket. In such environments any free haem is normally sequestered by albumin which is the major protein in gingival crevicular fluid [28] and by haemopexin, which play the important roles of restricting its bioavailability to bacteria. It is important to note that albumin also plays the role of removing haem from methaemoglobin [14], [15], [16], for the eventual transfer to haemopexin and hence to the liver for reprocessing. We therefore tested the ability of HmuY to compete with serum albumin for the haem in the form of methaemoglobin. Haem exchange experiments in which immobilised methaemoglobin-agarose was co-incubated with an equimolar amount of both albumin and HmuY, revealed the preferential rapid formation of the HmuY-ferrihaem complex. We also found that haem was preferentially bound by HmuY when it was introduced into a solution comprising equimolar amounts of both HmuY and albumin.

Of equal importance physiologically however, is the likelyhood that in a haem-limited environment in the absence of any free haem or of haemoglobin released from erythrocytes, HmuY may have to extract essential haem directly from host haem sequestering proteins. Accordingly, we tested whether HmuY could remove ferrihaem from methaemalbumin by co-incubating these two proteins in equimolar amounts. Spectroscopic analysis showed that loss of haem from the methaemalbumin and formation of a ferrihaem-HmuY complex occurred rapidly. This was confirmed by SDS-PAGE and TMB/H_2_O_2_ staining which showed that the haemalbumin was depleted in haem. Together, the above findings unequivocally demonstrated that HmuY can successfully compete with albumin for any available ferrihaem.

The K_a_ for HmuY and haem is of the order 3×10^2^ M^−1^ as determined by UV-visible and fluorescence analysis (Wojtowicz, H and Olczak, T, unpublished findings), whilst that for HmuR and haem is approximately 4×10^5^ M^−1^ [29]. It is thus easy to appreciate the facile haem transfer from HmuY to the HmuR receptor. However, it is noteworthy that the K_a_ of the albumin-haem binding system is around 10^8^ M^−1^ [20]. The pickup of haem by HmuY from methaemalbumin is therefore somewhat paradoxical from a simple consideration of the relative haem binding affinities of the two proteins. It is as yet unclear how haem exchange from albumin to HmuY is achieved, but it is likely that this may involve some other process such as induction in the change in molecular conformation of the albumin.

It is noteworthy that other bacterial haemophores have been shown to extract haem from methaemoglobin [30], [31]. However, it has been demonstrated here that the HmuY lipoprotein of *P. gingivalis* can extract haem not only from methaemoglobin, but also from deoxyhaemoglobin. We have also shown here for the first time that HmuY can remove haem from oxyhaemoglobin with the aid of specific haemoglobin degrading gingipain proteases. HmuY was also found to be capable of binding both free haem and that in present in methaemoglobin even in the presence of serum albumin. Given that albumin is the major protein component of gingival crevicular fluid and the periodontal pocket where it functions to sequester and withhold this essential nutrient from microbes, it is of significance that HmuY can also wrest haem from directly methaemalbumin. These findings further underline the versatility of the haemophore and protease systems of *P. gingivalis*, and demonstrate that *P. gingivalis* is highly capable of competing with the host in its primary habitats to obtain haem.

## Materials and Methods

### Purification of HmuY

*P. gingivalis* apoHmuY lacking the first 25 residues (NCBI accession number CAM 31898) was expressed using a pHmuY11 plasmid and *Escherichia coli* ER2566 cells (New England Biolabs) and purified from a soluble fraction of the *E. coli* lysate as previously described [7]. As the soluble protein released from the cell membrane, the recombinant HmuY lacked the signal peptide and first five amino acid residues (CGKKK) of the nascent secreted protein [7], [9].

### Gingipain purification

The arginine and lysine-specific gingipains HRgpA and Kgp, respectively, were isolated and purified from spent culture supernatants as previously described [26].

### Ferrihaem preparation

Iron(III) protoporphyrin IX was prepared as described previously [32], [28] by dissolving haemin (iron(III) protoporphyrin IX chloride, Fe(III)PPIX.Cl, at 1 mM in 0.1 M Tris, pH∼10, containing 0.14 M NaCl. The pH of this solution was then decreased to pH 7.5 by the slow addition of dilute HCl. The iron(II) protoporphyrin IX species was prepared from the above ferrihaem solution by reduction in the presence of 10 mM Na_2_S_2_O_4_ [2].

### Polyacrylamide gel electrophoresis (PAGE)

SDS-PAGE was carried out as previously described by Laemmli [33] and gels were stained for protein with Coomassie Brilliant Blue R-250 (CBB). Protein-bound haem was located on the gels by staining for haem-associated peroxidase activity using tetramethylbenzidine/H_2_O_2_ (TMB/H_2_O_2_), the samples being solubilised at 37°C for 1 h in Laemmli electrophoresis sample application buffer without dithiothreitol [17]. For native PAGE, urea and SDS were omitted from the separating gel, and the samples were also solubilised for 1 h in Laemmli sample application buffer without SDS, urea and dithiothreitol.

### Haem exchange experiments using immobilised haemoglobin

Bovine haemoglobin conjugated to agarose (Sigma Chemical Company Ltd.) was washed extensively in buffer (0.14 M NaCl, 0.1 M Tris-HCl, pH 7.5) to remove any free haem or un-conjugated haemoglobin as previously described [17]. The amount of haemoglobin conjugated per ml of agarose was determined from the UV-visible spectrum of the haemoglobin-agarose suspension. The spectrum revealed that the conjugated haemoglobin is in the oxidized form (methaemoglobin). The methaemoglobin-agarose beads were incubated at 37°C with HmuY (16 µM) and periodically pelleted by low speed centrifugation at 2000× *g* for 2 min and the UV-visible spectrum of the supernatant containing the HmuY was recorded. The supernatant solution was then added back to the beads which were then re-incubated as above prior to the next sampling.

### Haemoglobin preparations

Oxyhaemoglobin was prepared from fresh horse erythrocytes as previously described [19] and stored at -80°C in 0.14 M NaCl, 0.1 M Tris-HCl, pH 7.5. Methaemoglobin was prepared from the oxygenated protein by treatment with 64 µM NaNO_2_ in 0.14 M NaCl, 0.1 M Tris-HCl, pH 7.5 [12], [13] and stored in this buffer at −80°C until required. This yielded a preparation comprising 87% methaemoglobin as calculated from A_577 nm_ and A_630 nm_ values as described previously [12]. Proteolytically induced methaemoglobin was also prepared by treatment of oxyhaemoglobin with arginine-gingipain A (HRgpA) [13]. Briefly, oxyhaemoglobin (16 µM with respect to haemoglobin subunit) was pre-incubated with 0.4 µM HRgpA for 24 h at 37°C in 0.14 M NaCl, 0.1 M Tris-HCl, pH 7.5. This haemoglobin preparation comprised 77% methaemoglobin as calculated above.

Deoxyhaemoglobin-agarose was prepared by treating the haemoglobin agarose with 10 mM sodium dithionite, under an anaerobic atmosphere (80% nitrogen, 15% carbon dioxide and 5% hydrogen), in 0.14 M NaCl, 0.1 M Tris-HCl, pH 7.5. Once deoxygenated, the haemoglobin agarose beads (16 µM with respect to haemoglobin subunit) were incubated with an equi-molar amount of HmuY at 37°C under anaerobic conditions and at various times the beads were sedimented by centrifugation as above and the agarose-free supernatant solutions containing HmuY were sampled anaerobically, kept sealed in an optical cuvette and the spectrum recorded.

### Methaemalbumin preparation

Methaemalbumin was prepared by incubating a 120 µM stock solution of human albumin (Sigma product A-8763) at 37°C in 0.14 M NaCl, 0.1 M Tris-HCl, pH 7.5, with iron(III) protoporphyrin IX at a 1 ∶ 0.9 protein to haem molar ratio to ensure that no free uncomplexed haem remained in the preparation. The haemalbumin complex formed after 18 h displaying a Soret band at around 402 nm, along with bands at 500, 530 and 620 nm, in keeping with previous work [34].

### UV-visible spectroscopy

UV-visible spectra were recorded with a Ultrospec 2000 (Biochrom Ltd) using 1 cm pathlength cuvettes.
