# Correction: HmuY Haemophore and Gingipain Proteases Constitute a Unique Syntrophic System of Haem Acquisition by *Porphyromonas gingivalis*

**DOI:** 10.1371/annotation/8658727f-cc10-47ba-9c7e-8726f1f94b91

**Published:** 2011-03-04

**Authors:** John W. Smalley, Dominic P. Byrne, Andrew J. Birss, Halina Wojtowicz, Aneta Sroka, Jan Potempa, Teresa Olczak

The first affiliation is incorrect. It should read "Department of Clinical Infection, Microbiology and Immunology, Institute of Infection and Global Health, University of Liverpool, Liverpool, United Kingdom."

