# Correction: HmuY Haemophore and Gingipain Proteases Constitute a Unique Syntrophic System of Haem Acquisition by *Porphyromonas gingivalis*

**DOI:** 10.1371/annotation/919737a1-a459-4949-87ab-9fc56023a4cc

**Published:** 2011-03-04

**Authors:** John W. Smalley, Dominic P. Byrne, Andrew J. Birss, Halina Wojtowicz, Aneta Sroka, Jan Potempa, Teresa Olczak

There is an error in the third sentence of the funding section. The sentence should read: "J.W.S. is grateful to the School of Dentistry, University of Liverpool, for financial support for D.P.B."

